
JOURNAL INFORMATION
==============================
NLM Title Abbreviation: South Med Rec
NLM Journal ID: 0027317

The Southern Medical Record

Publisher: Biomedical Journal Digitization Project

ARTICLE INFORMATION
==============================
PMCID: PMC9089597
Article version: 1
Subjects: Editorial and Miscellaneous

Book Notices

Print publication date: 1879 Aug 20
Volume: 9
Issue: 8
First page: 318
Last page: 318
License: This work is free of known copyright restrictions. For more information, please see PMC Back Issue Digitization.
License URL: https://creativecommons.org/publicdomain/mark/1.0/

==============================

### BOOK NOTICES.

*A TREATISE ON THE HORSE AND HIS DISEASES*, by B. J. KENDALL. M.D., Enosburg Falls, Vermont.—88 pages, illustrated. A practical and useful work.

*PHARMACOPŒIA OF THE BRITISH HOSPITAL FOR DISEASES OF THE SKIN.*—London. Edited by BALMANO SQUIRE, M. B. LOUD, Senior Surgeon to the Hospital.—J & A. Churchill, publishers.

This is a very useful work to practitioners, especially to dermatologists.

*THE CLOCK STRUCK THREE*, being a review of "The Clock Struk One," and reply to it, showing the harmony between Christianity, Science and Spiritualism. Chicago: Religio-Philosophical Journal Publishing House, S. S. Jones, Proprietor. 352 pp. $1.50.

This work is by Rev. Samuel Watson, formerly a distinguished minister of the Methodist church, who claims, from a long and prayerful investigation of the subject, that the phenomena of modern Spiritualism are real, and will at no distant day, when purged of its follies and excresences, eventuate in the re-establishment of primitive Christianity as taught and exemplified by Christ and his Apostles, and is providentially designed to counteract and utterly to destroy the materialism and scientific infidelity of the day. The style of the writer, it must be conceded, is clear and forcible, and his views appear to flow from a sincere and earnest conviction. It would certainly be a happy consummation of tnis mysterious movement, should the views of Dr. Watson prove to be correct. But Spiritualists of his class can scarcely hope for a fair hearing so long as the mass of literature upon the subject continues to be directed against rather than in favor of the Bible and Christianity.

*ELEMENTS OF MODERN CHEMISTRY*, by ADOLPH WURTZ, Member of the Institute; Honorary Dean and Prof. of Chemistry of the Faculty of Medicine of Paris, etc.—Translated and edited with the approbation of the Author, from the fourth French edition, etc.; with 132 illustrations—676 pages—Philadelphia, J. B. Lippincott & Co., 1879.

This is a very valuable work in chemistry, embodying all the modern chemical advances, presented in a form of easy comprehension and acquirement. It is a translation of the fourth French edition of M. Wurtz, the great chemist of France—with changes and additions of interest to the American reader.

*POSTURE AS A MEANS OF RELIEF IN STRANGULATED AND INCARCERATED HERNIA*, with a General Consideration of the Mechanism of Reduction, by FRANK H. HAMILTON, A. M., M.D., Surgeon to Bellevue Hospital, etc.

### SPECIAL NOTICES.

**QUININE PILLS** of our make are prepared from Sulphate of Quinia of American manufacture, noted for its purity and excellence. Each Pill contains the full complement of material as expressed on the label, without the necessity of adding any excess to compensate for impurities. They are perfectly soluble, and physicians can depend upon obtaining the full benefit of the valuable remedy in a pleasant form. Always specify WARNER & Co., and avoid the substitution of cheaper and inferior brands.

**Lactated Pepsine.**—This preparation is composed of the following powerful digestive principles, of which each dose of five grains contains: Pepsine (pure), 1 grain (equal to 5 grains of saccharated pepsine); Pancreatine, ½ grain; Lactic Acid' ½ grain; Maltese, ¼ grain; Diastase, 1-15 grain; Hydrochloric Acid, 1-10 grain- Dose, 1 to 5 grains.

LACTATED PEPSINE will be found of great value in dyspepsia, all phases of indigestion, malnutrition, diarrhœa in infants, chronic diarrhœa, cholera infantum, constipation, and vomiting in pregnancy.

*Be particular to specify* "PARKE, DAVIS & Co.'s" LACTATED PEPSINE.

**SPEER'S Port Grape Wines for Weakly Persons.**—This excellent product of the grape is prescribed and used by the leading physicians in the country, and by the most fashionable families at evening entertainments, and by churches for communion. For sale by druggists.
